# Tacrolimus as an adjunct to autologous minced muscle grafts for the repair of a volumetric muscle loss injury

**DOI:** 10.1186/s40634-017-0112-6

**Published:** 2017-11-10

**Authors:** Benjamin T. Corona, Jessica C. Rivera, Joseph C. Wenke, Sarah M. Greising

**Affiliations:** Extremity Trauma and Regenerative Medicine Task Area, United States Army Institute of Surgical Research, 3698 Chambers Pass, BHT1, Fort Sam Houston, TX 78234 USA

**Keywords:** Neuromuscular strength, Orthopaedic trauma, Porcine, Skeletal muscle injury

## Abstract

**Background:**

Volumetric muscle loss (VML) following extremity orthopaedic trauma or surgery results in chronic functional deficits and disability. A current translational approach to address the devastating functional limitations due to VML injury is the use of an autologous minced muscle graft (~1 mm^3^ pieces of muscle tissue) replacement into the injured defect area, although limitations related to donor site morbidity are still unaddressed. This study was designed to explore adjunct pharmacological immunomodulation to enhance graft efficacy and promote muscle function following VML injury, and thereby reduce the amount of donor tissue required.

**Findings:**

Using a validated VML porcine injury model in which 20% of the muscle volume was surgically removed, this study examined muscle function over 3 months post-VML injury. In vivo isometric torque of the peroneus teritus (PT) muscle was not different before surgery among sham, non-repaired, non-repaired with tacrolimus, graft-repaired, and graft-repaired with tacrolimus VML groups. Bi-weekly torque analysis of the VML injured musculature presented a significant strength deficit of ~26% compared to pre-injury in the non-repaired, non-repaired with tacrolimus, and graft-repaired groups. Comparatively, the strength deficit in the graft-repair with systemic tacrolimus was marginally improved (~19%; *p* = 0.056). Both of the minced graft repaired groups presented a greater proportion of muscle tissue in full-thickness histology specimen.

**Conclusions:**

We demonstrate that adjunctive use of tacrolimus with an ~50% minced muscle graft replacement resulted in modest improvements in muscle function 3 months after injury and repair, but the magnitude of improvement is not expected to elicit clinically meaningful functional improvements.

## Introduction

Extremity orthopaedic trauma that results in volumetric muscle loss (VML) presents chronic and persistent functional muscle deficits, restricted joint range of motion, and fibrosis, which ultimately manifest as chronic disability (Mase et al. 2010; Corona et al. 2015; Garg et al. 2015; Rivera and Corona 2016). Moreover, VML injury may impair healing of concomitantly fractured bone (Willett et al. 2013; Hurtgen et al. 2016; Pollot et al. 2016; Hurtgen et al. 2017a, 2017b). In such cases where composite bone and skeletal muscle tissue injuries exist, clinical care emphasizes fracture healing with little attention to regeneration of the traumatized skeletal musculature. Recent basic and translational research efforts are aimed at promoting *de novo* regeneration of the muscle tissue to ameliorate musculoskeletal regeneration and to promote long-term improvements in muscle and limb function (Corona and Greising 2016).

In both small and large animal models treatment of VML injuries with autologous minced muscle grafts (~1 mm^3^ pieces of muscle tissue) have the ability to improve muscle function (Corona et al. 2013; Garg et al. 2014; Li et al. 2014; Aurora et al. 2015; Ward et al. 2015; Kasukonis et al. 2016; Ward et al. 2016). Autologous minced muscle grafts are capable of spontaneous *de novo* muscle fiber regeneration due to their composition of essential components to skeletal muscle regeneration, such as basal lamina and myogenic stem cells (Carlson 1972; Caldwell et al. 1990; Lepper et al. 2011). However, salient limitations of this approach for the repair of clinically relevant VML defects are the availability and morbidity associated with harvesting autologous muscle tissue, the rate of vascularization and innervation of regenerating muscle fibers, and the protracted pro-inflammatory wound environment that persists within VML injured skeletal muscle, which inhibits myogenic while activating fibrotic transcriptional programs (Hurtgen et al. 2016; Sadtler et al. 2016).

This study was designed to directly interrogate the impact of tacrolimus, an US Food and Drug Administration (FDA) approved immunomodulatory drug commonly used in transplant surgery, on functional regeneration mediated by autologous minced muscle graft repair of VML injury in a porcine model. Tacrolimus is a calcineurin phosphatase inhibitor that inhibits downstream Il-2 mediated activation of T lymphocytes and attenuates macrophage and dendritic cell activity (Thomson et al. 1995). Additionally, tacrolimus has neurotrophic properties that may accelerate axonal regeneration and muscle fiber reinnervation (Gold 1997; Sulaiman et al. 2002). As such, it was hypothesized that functional regeneration mediated by autologous minced muscle grafts would be augmented by adjunctive systemic delivery of tacrolimus.

## Methods

### Study design

Female Yorkshire Cross swine (*n* = 7) were purchased form Midwest Research Swine (Gibbon MN, USA). At the terminal experiment animals were 6.1 ± 0.2 months of age and considered to be young-adult (Table 1). All swine underwent initial functional testing and repeated testing every 2 weeks following surgery. At the time of surgery bilateral VML injuries (or sham procedures) were performed and then each limb was randomized to no repair or repair with a ~50% volume replacement using an autologous minced muscle graft harvested from the contralateral (i.e., non-repaired) limb (Ward et al. 2016). Immediately following surgery, each swine was further randomized to treatment groups, specifically 1 month of immunomodulation using tacrolimus or no treatment. The final functional data was examined at 12 weeks post-surgery and sampling of the muscles of the hindlimb was conducted. The primary assessment of these experimental groups was in vivo isometric torque, which was analyzed using a repeated measures approach. All protocols and animal care guidelines were approved by the Institutional Animal Care and Use Committee at the United States Army Institute of Surgical Research (Protocol #A14–018), in compliance with the Animal Welfare Act, the Implementing Animal Welfare Regulations and in accordance with the principles of the Guide for the Care and Use of Laboratory Animals.

**Table 1:** Characteristics across study period

	Sham	VML	VML + MMG	VML + Tacrolimus	VML + MMG + Tacrolimus
n=	1	2		4	
*Body mass (kg)*					
Week 0	39.4 ± 0.0	41.9 ± 2.3		41.7 ± 0.8	
Week 4	48.6 ± 0.0	49.9 ± 3.1		46.5 ± 0.7	
Week 8	54.2 ± 0.0	56.6 ± 2.8		51.8 ± 2.8	
Week 12	61.2 ± 0.0	65.3 ± 3.9		55.7 ± 3.3	
*Tacrolimus concentration (ng/ml)*					
Day 3	*nd*	*nd*		1.12 ± 0.29	
Day 9	*nd*	*nd*		1.35 ± 0.14	
Week 2	*nd*	*nd*		1.73 ± 0.25	
Week 3	*nd*	*nd*		2.26 ± 0.65	
Week 4	*nd*	*nd*		3.25 ± 1.58	
Week 6	*nd*	*nd*		*nd*	

Circulating levels of tacrolimus were analyzed; levels were non-detectible (*nd*) in the untreated and sham animals and 2 weeks following termination of treatment

Mean ± standard error

*VML* volumetric muscle loss, *MMG* minced muscle graft

### Surgical procedures

As previously described (Pollot and Corona, 2016; Greising et al. 2017), swine were anesthetized with Telazol (4–6 mg/kg, i.m.), intubated and maintained under isoflurane anesthesia. Under appropriate aseptic conditions, a 10 cm incision was made centered over the anterior compartment to expose the peroneus tertius (PT) muscle. The anterior aspect of the muscle was measured and a 3 × 3 cm^2^ area was marked in the middle third of the PT muscle. Then this area of muscle (5.59 ± 0.04 g) was sharply dissected from the PT muscle. Sham operated limbs were surgically exposed and the PT muscle was isolated, but no tissue was removed. A subset of VML defects were treated with autologous minced muscle grafts as previously described (Pollot et al. 2016; Ward et al. 2016). Briefly using the muscle from the contralateral VML injury an autologous minced muscle graft was created. Under sterile conditions the muscle was minced to ~1 mm^3^ pieces and ~50% volume was transplanted into the defect of the designated repair limb. To accommodate the autograft into the defect, the epimysium layer superficial to the muscle was closed over autograft with ~8 interrupted proline sutures. In all cases the fascial and skin incisions were closed in layers with absorbable suture and compressive bandages were wrapped around each limb. Post-surgical analgesic administration of Buprenorphine SR (0.01 mg/kg, s.c.), Rimadyl (4.4 mg/kg, s.c.), and Excede (5.0 mg/kg, s.c.) was conducted through 1 week post-surgically.

### Immunomodulatory treatment

For the first month following surgery animals randomized to the tacrolimus immunomodulation group were administered 0.075 mg/kg daily (Prograft, Astellas Pharma Inc.; Northbrook IL, USA). Daily administration ranged from 3 to 4 mg during the treatment period to account for body weight; all treated and non-treated swine gained approximately the same amount of weight per week. The selected dosage is recommended for cardiac transplant surgery and has been shown to result in effective serum levels of tacrolimus of 10–20 ng/ml (Podesser et al. 2005); notably whole blood concentrations can be variable as treatment begins. Evaluation of the whole blood concentrations of tacrolimus was completed by HPLC-tandem mass spec at day 3, 9, 14, 28, and 42 post-surgery, using blood collected in EDTA anti-coagulant coated tubes. No adverse events related to tacrolimus treatment were observed.

### In vivo functional testing

As previously described and validated (Ward et al. 2016; Greising et al. 2017), the strength of the dorsiflexor muscles of the anterior compartment was by subdermal stimulation of the peroneal nerve using needle electrodes over a range of joint angles (0–50^o^). Maximal isometric tetanic torque was elicited using 100 Hz, 0.1 ms pulse, over 800 ms (Grass S88 stimulator and 890A Aurora Scientific; Ontario Canada). Torque was normalized to body weight assessed immediately prior to each procedure.

### Histological analyses

The entire PT muscle was excised and muscle samples were subsequently excised using an 8 mm biopsy punch through the full thickness of the muscle, following the terminal in vivo functional assessment. Muscle sections were frozen in melting isopentane and stored at -80 °C until processing and analysis. Sections were treated processing using standard histologic procedures, with each section being cross-sectioned at 8 μm and stained using Masson’s trichrome. Bright field images were acquired with a Zeiss Axio Scan.Z1 (Carl Zeiss Microscopy, Thornwood NY, USA) and composite images were saved separately as a 24-bit, 96 dpi color images. For display purposes only images were produced in Adobe Photoshop (Adobe Systems Inc.; San Jose CA, USA) by down-converting, without introducing any changes in brightness or contrast. During all imaging and investigation, investigators were blinded to experimental group.

### Statistical analyses

All data was analyzed using JMP (version 10.0 SAS Institute, Inc., Cary NC, USA). The primary assessment (isometric torque) was analyzed using repeated measures ANOVA. Blood levels of tacrolimus were analyzed using one way-ANOVA. When appropriate Tukey HSD post-hoc analysis was performed. Data are reported as mean ± standard error, and significance was determined at the α < 0.05 level.

## Findings

All animals recovered promptly following surgery and within 24 h displayed normal mobility and cage activity. Additionally no adverse events occurred for the duration of the study. Circulating whole blood levels of tacrolimus in treated animals was ~3.3 ng/ml by 4 weeks post-surgery and was no longer detectible 2 weeks later (Table 1).

### PT muscle histology

**Fig. 1:**
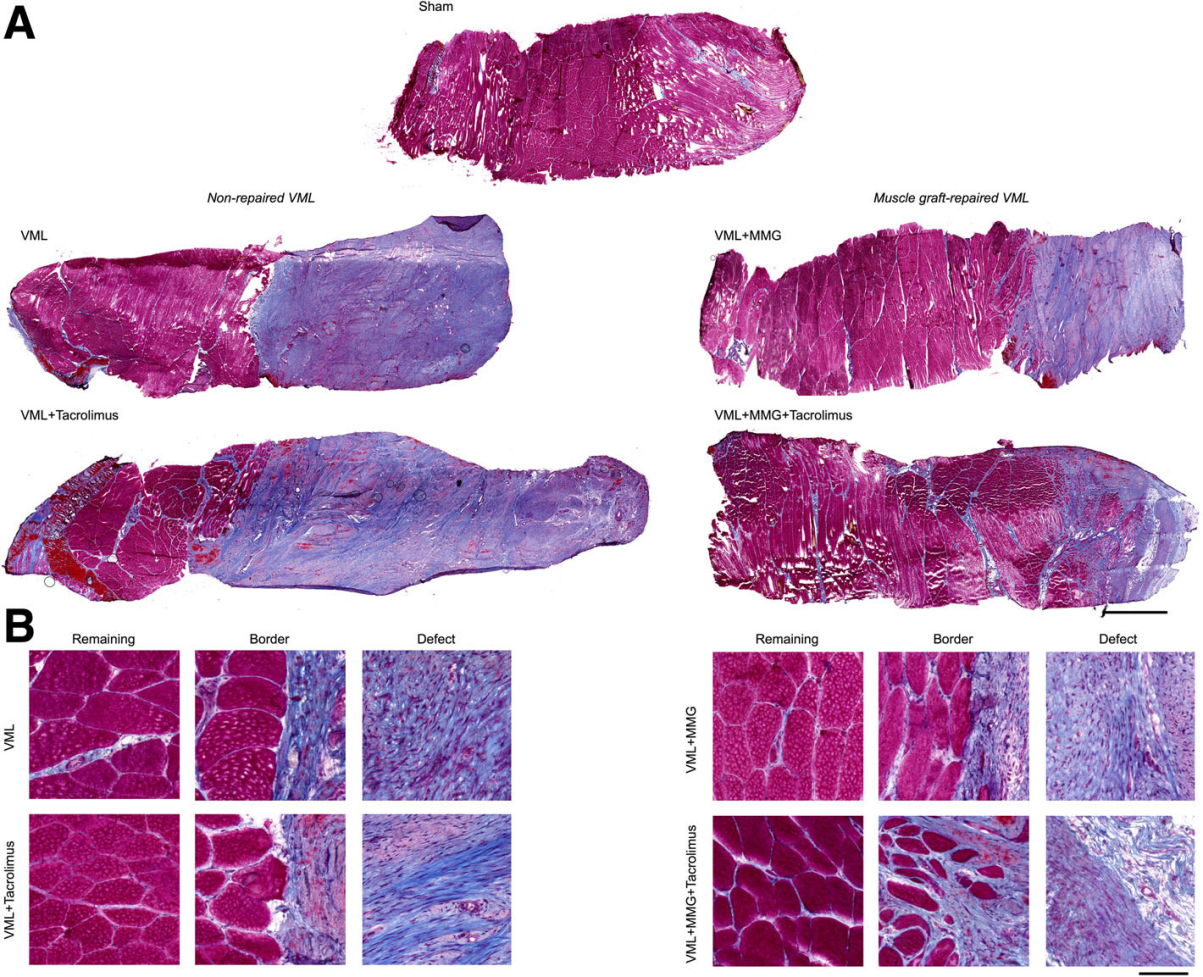
Representative histologic micrographs of Masson’s Trichrome stained (connective tissue is blue; nuclei are purple; skeletal muscle fibers are red) porcine peroneous tertius (PT) muscle following VML injury and repair. While all PT muscles indicate gross fibrosis following VML injury, only the muscle graft-repaired displayed areas of likely regenerated fibers. There were no apparent differences due to treatment with tacrolimus. (**a)** Each sample represents a full thickness sample through the muscle. Scale bar is 2 mm; all images are at the same magnification. **(b)** Representative inserts from the remaining muscle, border, and defect area of the samples are displayed. Scale bar is 100 μm; all images are at the same magnification

Histological examination of the PT muscle indicated significant fibrotic deposition overlaying the remaining muscle across all treatment groups (Fig. 1). The fibrous tissue was comprised of a mixture of immature and mature connective tissue matrix, with the more mature fibrosis indicated by dense collagen fibers with trabecular meshwork. In all cases the muscle remaining after VML injury appeared healthy, without noteworthy signs of degeneration. Sections from PT muscles that were repaired with a muscle graft presented noticeably lesser fibrotic tissue and greater muscle tissue than non-repaired VML injured muscles, regardless of tacrolimus delivery.

### In vivo anterior crural muscle strength

**Fig. 2:**
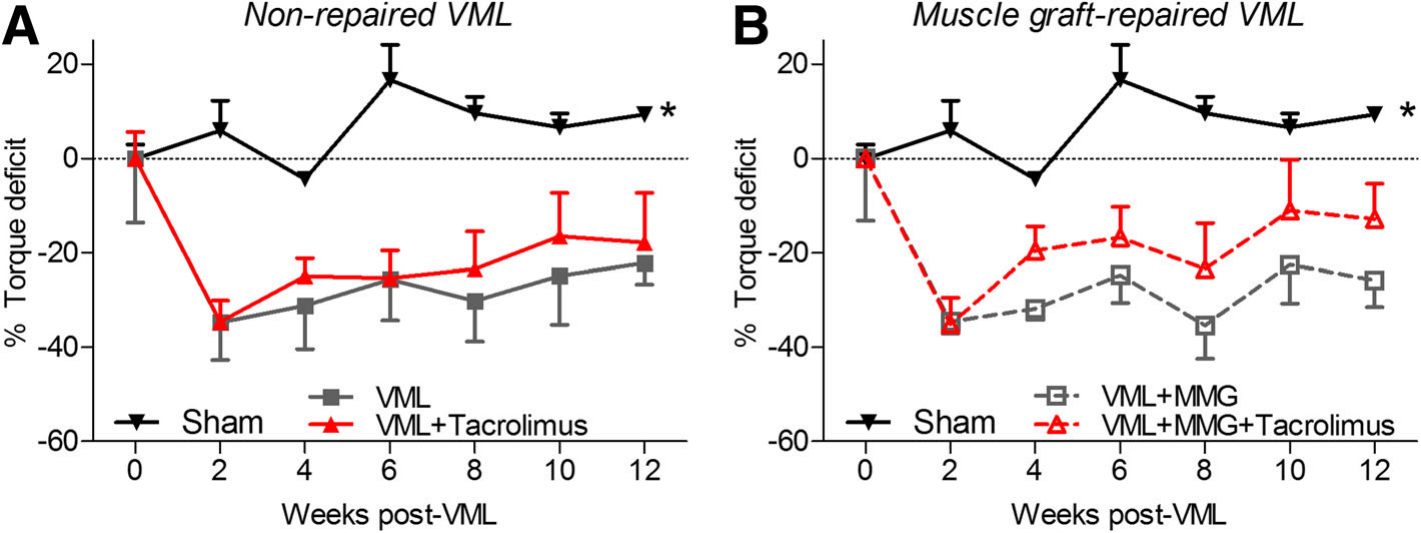
Peroneus teritus (PT) muscle function was determined by peroneal nerve stimulation *in vivo*. The percent torque deficit to the pre-injury torque was determined for the (**a**) non-repaired and (**b**) muscle graft (MMG) repaired groups following VML injury and the effect of adjunctive tacrolimus was investigated across these groups. The strength of the sham operated group was consistent over time (duplicated in each panel) and all VML injured groups were less than sham operated. All data analyzed by two-way ANOVA (group *p* < 0.001; time *p* = 0.104; interaction *p* = 0.999). * Significant main effect of group, sham > all other surgical groups. Data are mean ± standard error

Repeated evaluation of maximally stimulated peak isometric torque of the anterior compartment was conducted immediately before and every 2 weeks following VML injury by maximally stimulating the peroneal nerve (Fig. 2). Prior to surgery, isometric torque was not different among groups (*p* = 0.542). As expected, sham-operated limbs presented consistent torque production over the 12 week study (*p* = 0.121). Non-repaired (i.e., no minced grafts) VML-injured muscles presented an average ~28% isometric torque deficit from 2 to 12 weeks post-injury, which was not ameliorated by tacrolimus administration (~24% torque deficit). Similarly, minced graft repair without tacrolimus presented a ~29% functional deficit. However, the inclusion of tacrolimus delivery with minced graft repair reduce the functional deficit by approximately one-third to ~19%. The functional deficit for the minced graft with tacrolimus trended to be statistically different from non-repaired VML injured muscle without tacrolimus (*p* = 0.088) and minced muscle graft repaired VML injured muscle (*p* = 0.056).

## Discussion

We demonstrate that adjunct tacrolimus treatment to autologous minced muscle grafts tended to ameliorate neuromuscular strength deficits following VML injury. However, the data do not support a clinically meaningful therapeutic benefit using the current dosing regimen. Autologous minced muscle grafts provide necessary endogens elements of skeletal muscle necessary for repair, importantly including satellite cells. This approach does not require FDA approval as the grafts are minimally manipulated tissue. However, a current clinical limitation of autologous minced muscle grafts is the donor site burden; as such we investigated a more clinically feasible replacement volume.

Herein we delivered systemic tacrolimus for 1 month following VML injury, during a period in which myogenesis is expected to be increased. The target dosage of systemic tacrolimus was selected as it has been previously used for successful cardiac transplant (Podesser et al. 2005). While the circulating concentrations of tacrolimus were slightly below the expected range it is still expected that systemic delivery of tacrolimus presumably suppressed the immune response within the VML injured muscle. Given the exacerbated immune response that follows VML injury (Hurtgen et al. 2016) and the known detrimental impact that prolonged inflammation has on myogenesis (Tidball 2005), the original hypothesis that immunomodulation can ameliorate functional regeneration remains plausible. Though, clearly further testing is necessary to identify either an effective VML-injury specific dosing regimen for tacrolimus or a different drug that more specifically targets influential targets of the immune response to VML injury. Towards that end, heightened and prolonged macrophage presence and pro-inflammatory activity has been observed in rodent trauma models that involve VML injury (Hurtgen et al. 2016; Hurtgen et al. 2017a, 2017b), and thus investigation of targeted macrophage modulation may prove effective.

It was unexpected that the autologous minced muscle graft repair alone did not improve muscle strength. Previously, a ~100% autologous minced muscle graft repair of VML injury that resulted in ~32% strength increase compared to non-repaired VML injuries in a large animal model (Ward et al. 2016). In the current study, only 50% of the mass of the VML defect was replaced with an autologous minced muscle grafts and therefore it may be that in this large animal model a lower threshold for repair was crossed. Prior observations in which a 50 and 100% minced graft repair achieved similar levels of muscle fiber regeneration and strength recovery in a rat tibialis anterior muscle VML model supported the use of this level of repair in the current study. Somewhat encouraging, however, was the obvious greater proportion of skeletal muscle tissue in the minced graft repair versus the non-repaired groups. It is expected that this proportion of muscle fibers is due to *de novo* regeneration of fibers, which has been previously validated in rodent models (Ward et al. 2015; Corona et al. 2017). It is possible that the minced grafts did indeed promote skeletal muscle regeneration but that the fibers were not yet innervated. In that scenario, the tendency for improved strength in the minced graft group with adjunct tacrolimus administration may reflect the compounds (i.e., FK506) beneficial impact on peripheral nerve regeneration (Gold 1997; Sulaiman et al. 2002).

## Conclusion

The current study demonstrates that delivery of tacrolimus as an adjunct to autologous minced graft repair of VML injury has the potential to reduce prolonged muscle functional deficits. Notably, delivery of ~50% minced grafts to the VML without adjunctive immunomodulation did not promote recovery of strength, but did appear to promote muscle tissue regeneration. These findings highlight the need for further studies to delineate an effective immunomodulatory approach to augment minced muscle grafts in the repair of VML injury; such studies may be best suited to a standardized rodent VML model.

## Abbreviations

FDA: US Food and Drug Administration
PT: Peroneus teritus
VML: Volumetric muscle loss
